# Comprehensiveness, Accuracy, and Readability of Exercise Recommendations Provided by an AI-Based Chatbot: Mixed Methods Study

**DOI:** 10.2196/51308

**Published:** 2024-01-11

**Authors:** Amanda L Zaleski, Rachel Berkowsky, Kelly Jean Thomas Craig, Linda S Pescatello

**Affiliations:** 1 Clinical Evidence Development Aetna Medical Affairs CVS Health Corporation Hartford, CT United States; 2 Department of Preventive Cardiology Hartford Hospital Hartford, CT United States; 3 Department of Kinesiology University of Connecticut Storrs, CT United States

**Keywords:** exercise prescription, health literacy, large language model, patient education, artificial intelligence, AI, chatbot

## Abstract

**Background:**

Regular physical activity is critical for health and disease prevention. Yet, health care providers and patients face barriers to implement evidence-based lifestyle recommendations. The potential to augment care with the increased availability of artificial intelligence (AI) technologies is limitless; however, the suitability of AI-generated exercise recommendations has yet to be explored.

**Objective:**

The purpose of this study was to assess the comprehensiveness, accuracy, and readability of individualized exercise recommendations generated by a novel AI chatbot.

**Methods:**

A coding scheme was developed to score AI-generated exercise recommendations across ten categories informed by gold-standard exercise recommendations, including (1) health condition–specific benefits of exercise, (2) exercise preparticipation health screening, (3) frequency, (4) intensity, (5) time, (6) type, (7) volume, (8) progression, (9) special considerations, and (10) references to the primary literature. The AI chatbot was prompted to provide individualized exercise recommendations for 26 clinical populations using an open-source application programming interface. Two independent reviewers coded AI-generated content for each category and calculated comprehensiveness (%) and factual accuracy (%) on a scale of 0%-100%. Readability was assessed using the Flesch-Kincaid formula. Qualitative analysis identified and categorized themes from AI-generated output.

**Results:**

AI-generated exercise recommendations were 41.2% (107/260) comprehensive and 90.7% (146/161) accurate, with the majority (8/15, 53%) of inaccuracy related to the need for exercise preparticipation medical clearance. Average readability level of AI-generated exercise recommendations was at the college level (mean 13.7, SD 1.7), with an average Flesch reading ease score of 31.1 (SD 7.7). Several recurring themes and observations of AI-generated output included concern for liability and safety, preference for aerobic exercise, and potential bias and direct discrimination against certain age-based populations and individuals with disabilities.

**Conclusions:**

There were notable gaps in the comprehensiveness, accuracy, and readability of AI-generated exercise recommendations. Exercise and health care professionals should be aware of these limitations when using and endorsing AI-based technologies as a tool to support lifestyle change involving exercise.

## Introduction

Regular physical activity is an essential component of a healthy lifestyle with numerous benefits that are widely recognized and indisputable [1,2]. To support overall health, the American College of Sports Medicine (ACSM) and the Department of Health and Human Services recommend healthy adults engage in regular physical activity, including moderate-intensity aerobic exercise for at least 150 minutes per week, vigorous-intensity aerobic exercise for at least 75 minutes per week, or a combination of both, as well as muscle-strengthening activities at least twice per week [1,2]. In addition, evidence-based practice calls for exercise as first-line therapy to prevent, treat, and control multiple chronic conditions and diseases such as hypertension, hypercholesterolemia, and diabetes mellitus [3-7]. As such, the ACSM endorses individualized, evidence-based, exercise recommendations (termed *exercise prescription* [ExRx]) for more than 25 clinical populations [1]. These ExRxs are tailored to favorably augment health-related outcomes of interest for each respective clinical population while addressing additional factors such as clinical contraindications, common medications, and special considerations [1,8]. Despite well-established guidelines, health care providers often struggle to provide sufficient counseling and follow-up on lifestyle recommendations, including exercise, due to various barriers such as time constraints, limited resources, lack of awareness or training, and lack of reimbursement incentives [9-11]. Patients also rely heavily on web-based sources for health-related information [12-14], which often includes misinformation that can negatively impact health outcomes and undermine provider-led efforts to support behavior change [15,16].

Artificial intelligence (AI) has recently emerged as a promising tool to augment health and health care and address these challenges [17]. AI-based technology including machine learning, neural networking, deep learning, and natural language processing enables computers to interact with a corpus of text data to generate human language [18,19]. Large language models (LLMs), such as the generative pretrained transformer (GPT), have the ability to generate human-like language on their own, making them a powerful tool for interacting with users as if they are communicating with another human [18,19]. The surge in popularity of LLMs can largely be attributed to the third iteration of OpenAI’s GPT series, ChatGPT [20]. ChatGPT has been recognized as the fastest-growing consumer application in history [20] and is widely regarded as disruptive technology due to its strong potential to enable a wide range of clinical applications as both a provider- and patient-facing tool [21] by generating language that is contextually appropriate, natural sounding, and coherent. Indeed, ChatGPT has demonstrated remarkable capabilities including diagnosis support, streamlining clinical workflows, reducing documentation burden, improving patient education understandability and experience [22-25], and, most recently, passing the United States Medical Licensing Examination [26].

Transformative applications of ChatGPT continue to evolve, but evaluation of its output and suitability in clinical context remains to be explored, in addition to identifying barriers to access and outcomes related to its use. The application of digital technology to support a health behavior change using knowledge-shaping techniques, which is complex and riddled with contextual and individualized components, is challenging [27]. Challenges include ensuring the suitability and usability of the technology confers appropriate educational requisites to understand and apply knowledge in the form of its recommendations. These educational considerations include readability, which can influence the use of AI-generated education for health behavior change [28]. Further, as an extension of readability, low health literacy can limit a patient's ability to understand and use health information effectively, which can reduce the effectiveness of AI-generated educational resources [29,30].

The evaluation of ChatGPT’s suitability to provide interactive, personalized, and evidence-based exercise recommendations to support behavior change to improve health has not been conducted to date. As such, the primary aim of this study is to assess the suitability of exercise recommendations generated by ChatGPT, a new AI chatbot, as an adjuvant educational tool for health care providers and patients. Primary outcomes of interest include comprehensiveness, accuracy, and readability of the recommendations generated by ChatGPT, with the goal of determining its potential to deliver personalized exercise recommendations at scale. A secondary aim of this study was to conduct a qualitative analysis to identify potential patterns, consistencies, and gaps in AI-generated exercise recommendations. As this technology is still nascent, the study was exploratory in nature, without an a priori hypothesis.

## Methods

### High-Level Overview

**Figure 1:**
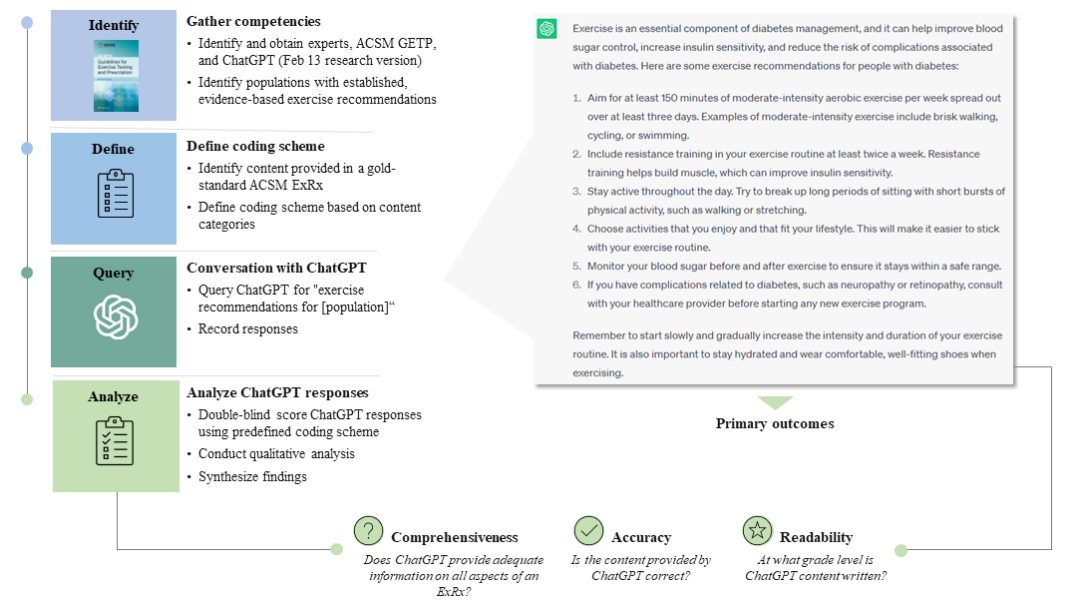
Conceptual study overview. ACSM: American College of Sports Medicine; ExRx: exercise prescription; GETP: Guidelines for Exercise Testing and Prescription.

This study was conducted in March 2023 using the free research preview of a novel AI chatbot (ChatGPT February 13 version) [31]. Figure 1 provides a conceptual overview of the study. Briefly, open-text queries seeking individualized exercise advice were posed to the chatbot interface for all populations (N=26) for which there exist established evidence-based exercise recommendations by the ACSM [1]. Mixed methods were applied to characterize individual and average exercise recommendation content depth, accuracy, and readability. The results were synthesized to highlight potential strengths, weaknesses, opportunities, and risks for researchers, clinicians, and patients likely to interact with the ChatGPT platform for this use case.

### Ethical Considerations

This study was deemed to be exempt by the University of Connecticut Institutional Review Board (E23-0378) as this study solely involved the evaluation of AI-generated output and did not involve interaction or intervention with human subjects.

### Selection of the Gold-Standard Reference Source

The ACSM is widely regarded as a leading authority in the field of exercise science and sports medicine, and the organization’s guidelines and recommendations are considered the gold standard for health and fitness professionals in the United States and the world [1,8,32]. The *ACSM’s Guidelines for Exercise Testing and Prescription* (GETP) serves as its flagship resource manual, continuously updated every 4-5 years since 1975. The most recent edition integrates the latest guidelines from ACSM position stands and other relevant professional organizations' scientific statements, including the 2018 Physical Activity Guidelines for Americans [1]. This latest edition of GETP represents the most current and primary resource for evidence-based exercise recommendations [1]. Given ACSM's authoritative status and the comprehensiveness of its guidelines, GETP was selected as the ground truth benchmark source to guide the study design and systematically evaluate the suitability of AI-generated exercise recommendations.

### ChatGPT Prompt Specificity and Structure

Prompt methodology was developed a priori with the overarching goal to observe ChatGPT’s unaltered performance in a real-world setting while controlling for factors known to influence output, including prompt structure, evaluation timeframe, model version, and model feedback.

A single researcher (ALZ) posed 26 separate, open-ended prompts as a new chat session to the ChatGPT bot (version 3.5) on the same day in a single session. Each text prompt was framed to the ChatGPT bot in a standardized, neutral, third-person tense format as “exercise recommendations for [population]” to optimize the relevance of AI responses for both health care provider and patient scenarios. Generated ChatGPT bot responses were abstracted from the interface and converted into plain text format using Microsoft Word (version 2208; Microsoft Corp) on the same day. Content was unaltered upon conversion to plain text format (Multimedia Appendix 1). Note that the ChatGPT bot used in this study was not subjected to retraining or correction during these prompt interactions. The rationale for this methodological decision was to enable the natural observation of ChatGPT’s raw performance and provide a transparent evaluation of its inherent capabilities [33,34].

### AI-Generated Exercise Recommendations

Following this prompt specificity and structure, all clinical populations within the ACSM GETP were evaluated once in a separate prompt (N=26), including healthy adults, children and adolescents, older adults, persons who are pregnant, and individuals with cardiovascular disease (CVD), heart failure, heart transplant, peripheral artery disease, cerebrovascular accident, asthma, chronic obstructive pulmonary disease, diabetes mellitus, dyslipidemia, hypertension, overweight and obesity, arthritis, cancer, fibromyalgia, HIV, kidney disease, multiple sclerosis, osteoporosis, spinal cord injury, Alzheimer disease, intellectual disability, and Parkinson disease.

### Conceptual Content Analysis

A list of conceptual categories was generated, refined, and organized into a coding scheme for predefined categories that pertain to the fundamental aspects of an ExRx. These categories relate to an individualized physical activity program based on the FITT principle, which stands for the frequency (*how often?*), intensity (*how hard?*), time (*how long?*), and type (*what kind?*) of exercise [1,35]. The final coding scheme included ten categories: (1) health condition–specific benefits of exercise, (2) exercise preparticipation health screening, (3) frequency, (4) intensity, (5) time, (6) type, (7) volume, (8) progression, (9) special considerations, and (10) references (ie, citations to primary literature or sources that supported the AI-generated content provided).

AI-generated exercise recommendations were then coded and recorded in Microsoft Excel (version 2208; Microsoft Corp) following a 2-stage coding process by 2 independent coders with advanced degrees in kinesiology (ALZ and RB). In the first stage, AI-generated content was appraised for comprehensiveness. Each exercise recommendation was coded for the presence (1 point) or absence (0 points) of content provided for each of the 10 prespecified categories such that each exercise recommendation had a possible range of 0-10 points. Comprehensiveness was determined by dividing the total number of points (ie, *actual*) by the total number possible (ie, *expected* or 10 points) and multiplying by 100. The resulting score was expressed as a percent, with 100% indicating the highest possible score and fully comprehensive. This formula was applied to all 26 exercise recommendations and averaged to characterize ChatGPT’s overall ability to deliver exercise recommendations regarding their comprehensiveness.

In the second stage, all categories with reported content (ie, fully *and* partially comprehensive content) were appraised for accuracy. Accuracy was defined as concordance with the ACSM GETP as the ground truth source [1]. In one instance, content deviated from the ACSM GETP (ie, condition-specific benefits of exercise for individuals with HIV), and accuracy was defined as the degree to which the content was consistent with other widely established facts or clinical literature. Responses were coded by the same independent reviewers (ALZ and RB) and recorded as binary variables: “concordant” or “discordant” following the same process used to determine comprehensiveness. Potential discrepancies in coding were resolved through discussion with a third party and senior expert in the field (LSP). The accuracy score was determined by dividing the number of concordant category counts by the number of categories present (ie, “actual” counts; previously determined when calculating comprehensiveness during the first stage) and multiplying by 100. The resulting score was expressed as a percent, with 100% indicating the highest possible accuracy score or fully concordant.

### Readability Metrics

The Flesch-Kincaid formula was used to determine readability, a commonly used tool that evaluates the complexity of text-based educational material. This tool was selected due to its objectivity, as scores are computationally derived rather than paper-and-pencil tools that rely on hand calculations and subjectivity, which introduce risk for human error [36]. The formula is based on the average number of syllables per word and the average number of words per sentence with the resulting score estimating the minimum grade level required to understand the text. For example, a score of 8.0 means that the text can be understood by an average eighth-grader in the United States. Flesch reading ease scores range from 0 to 100, with higher scores indicating easier-to-read text. For example, scores <50 are considered difficult to read, while scores >80 are considered easy to read [36]. To assess readability metrics and word count, a single researcher (RB) used the built-in readability statistics functionality of Microsoft Word (version 2208). The mean (SD) word count and readability metrics (ie, Flesch reading ease and grade level) were calculated using Microsoft Excel (version 2208).

### Qualitative Analysis

Qualitative analysis with a thematic mapping approach was used to identify novel patterns, trends, and insights across the AI-generated text output. Thematic mapping, a qualitative research method, involves the identification, analysis, and visualization of recurring themes or topics within a data set. This approach is instrumental in highlighting consistencies or gaps in data, facilitating the generation of insights, and formulating hypotheses for further investigation [37].

### Statistical Analyses

Descriptive statistics characterized the distribution of all outcome variables of interest, including comprehensiveness, accuracy, and readability metrics. Interrater reliability was assessed using Cohen κ coefficient [(observed agreement–expected agreement)/(1–expected agreement)]. Qualitative analysis was conducted using a systematic multistep approach. All AI-generated exercise recommendations, comprising the text output, were collected and organized to form the data set for qualitative examination. The analysis was carried out by a single researcher (ALZ) who immersed themselves in the content and initiated the coding process by identifying initial themes or patterns within the recommendations. Subsequently, codes were meticulously refined and organized into broader themes, ensuring consistency and accuracy throughout the process. These identified themes were then visually mapped to represent patterns within the data set. Insights generated from the analysis were discussed collaboratively as a team, facilitating comprehensive understanding and quantification, whenever applicable.

## Results

### Interrater Reliability

Interrater reliability was assessed for the 2 independent raters who coded a sample of 26 AI-generated exercise recommendations using a set of 10 categories. Cohen κ coefficient was calculated to be 1.0, indicating perfect agreement between coders.

### Comprehensiveness of AI-Generated Exercise Recommendations

Table 1 details the presence of educational content across the predefined categories of interest abstracted from AI-generated exercise recommendations for 26 populations. Overall, AI-generated exercise recommendations were 41.2% (107/260) comprehensive when compared against a predefined set of content categories that comprise a gold-standard ExRx [1]. There were no populations or categories that were fully comprehensive. Comprehensiveness ranged from 0% to 92% with notable gaps in content surrounding the critical components of ExRx: frequency (n=2, 8%), intensity (n=2, 8%), time (n=1, 4%), and volume (n=0, 0%). Partial information was provided across these same categories (ranging from 31% to 58%) with almost all gaps surrounding the provision of FITT for resistance training or flexibility modalities. In addition, only 8% (n=2) of recommendations provided a reference source, both of which (accurately) cited the American Heart Association.

**Table 1:** Comprehensiveness of artificial intelligence–generated exercise recommendations by content category (N=26).

Content	Exercise recommendations reporting content		
	Fully provided, n (%)	Partial^a^, n (%)	Not provided, n (%)
Condition-specific benefits	24 (92)	0 (0)	2 (8)
Preparticipation screening	24 (92)	0 (0)	2 (8)
Frequency	2 (8)	9 (35)	15 (58)
Intensity	2 (8)	15 (58)	9 (35)
Time	1 (4)	10 (38)	15 (58)
Type	14 (54)	12 (46)	0 (0)
Volume	0 (0)	8 (31)	18 (69)
Progression	15 (58)	0 (0)	11 (42)
Special considerations	23 (88)	0 (0)	3 (12)
References	2 (8)	0 (0)	24 (92)

^a^Partial indicates some, but not all, possible content was provided.

### Accuracy of AI-Generated Exercise Recommendations

Of the total available content provided to the end user, AI-generated exercise recommendations were 90.7% (146/161) accurate when compared to a gold-standard reference source (ie, ACSM GETP [1]). Among the 9.3% (15/161) of inaccurate recommendations (Table 2), there were 15 counts of discordance with most misinformation counts (n=8, 53%) surrounding the need for preparticipation medical clearance prior to engaging in exercise. The second highest category of discordance was within education related to frequency (n=2, 13%) with “overprescribing” aerobic exercise for Alzheimer disease and fibromyalgia by 2 and 5 days per week, respectively. There was 1 count each of discordance across 5 content categories (ie, condition-specific benefits, intensity, time, type, and progression) and 0 counts of discordance across the remaining content categories, including volume, special considerations, and references.

When comparing populations with discordance, hypertension (n=3) had the greatest number of misinformation counts followed by individuals with fibromyalgia (n=2), healthy adults (n=1), older adults (n=1), and cancer (n=1) and individuals with Alzheimer disease (n=1), arthritis (n=1), dyslipidemia (n=1), HIV (n=1), multiple sclerosis (n=1), osteoporosis (n=1), and overweight and obesity (n=1).

**Table 2:** Summary of inaccurate content among AI^a^-generated exercise recommendations for all reported content categories (N=161).

Content category (count/reported) and population		AI-generated content	Ground truth
**Condition-specific benefits (1/24)**			
	HIV	“Regular exercise can be beneficial for people living with HIV. It can help improve cardiovascular health, reduce stress and anxiety, boost immune function, and enhance overall well-being.”	Research regarding the impact of exercise to improve immune function is inconclusive [38].
**Preparticipation screening (8/24)**			
	Healthy adult	“Remember to always listen to your body and consult with a healthcare professional before starting a new exercise routine.”	ACSM^b^ preparticipation screening guidelines state that “physically inactive but otherwise healthy asymptomatic persons may begin light- to moderate-intensity exercise without medical clearance and, in the absence of symptoms, progress gradually in intensity as recommended by current ACSM exercise prescription guidelines” [1].
	Older adult	“However, it's important for older adults to choose exercises that are appropriate for their age and fitness level, and to check with their doctor before starting a new exercise program.”	ACSM preparticipation screening guidelines state that “physically inactive but otherwise healthy asymptomatic persons may begin light- to moderate-intensity exercise without medical clearance and, in the absence of symptoms, progress gradually in intensity as recommended by current ACSM exercise prescription guidelines” [1].
	Dyslipidemia	“It's important to consult with a healthcare professional before starting a new exercise program ...”	ACSM preparticipation screening guidelines state that “physically inactive but otherwise healthy asymptomatic persons may begin light- to moderate-intensity exercise without medical clearance and, in the absence of symptoms, progress gradually in intensity as recommended by current ACSM exercise prescription guidelines” [1].
	Hypertension	“Consult with your doctor: Before starting any exercise program, it is important to consult with your doctor to make sure it is safe for you to exercise and to get guidance on the most appropriate type and intensity of exercise.”	ACSM preparticipation screening guidelines state that “physically inactive but otherwise healthy asymptomatic persons may begin light- to moderate-intensity exercise without medical clearance and, in the absence of symptoms, progress gradually in intensity as recommended by current ACSM exercise prescription guidelines” [1].
	Arthritis	“Be sure to talk to your doctor or a physical therapist before starting any new exercise program ...”	ACSM preparticipation screening guidelines state that “physically inactive but otherwise healthy asymptomatic persons may begin light- to moderate-intensity exercise without medical clearance and, in the absence of symptoms, progress gradually in intensity as recommended by current ACSM exercise prescription guidelines” [1].
	Cancer survivors	“It's important to work with a healthcare provider or a qualified exercise professional to develop a safe and effective exercise program that meets your individual needs and abilities.”	ACSM preparticipation screening guidelines state that “physically inactive but otherwise healthy asymptomatic persons may begin light- to moderate-intensity exercise without medical clearance and, in the absence of symptoms, progress gradually in intensity as recommended by current ACSM exercise prescription guidelines” [1].
	Multiple sclerosis	“It is always recommended to consult with a healthcare professional before starting any exercise program.”	ACSM preparticipation screening guidelines state that “physically inactive but otherwise healthy asymptomatic persons may begin light- to moderate-intensity exercise without medical clearance and, in the absence of symptoms, progress gradually in intensity as recommended by current ACSM exercise prescription guidelines” [1].
	Osteoporosis	“It's important to talk to your doctor or a qualified exercise professional before starting any new exercise program, especially if you have osteoporosis or other medical conditions.”	ACSM preparticipation screening guidelines state that “physically inactive but otherwise healthy asymptomatic persons may begin light- to moderate-intensity exercise without medical clearance and, in the absence of symptoms, progress gradually in intensity as recommended by current ACSM exercise prescription guidelines” [1].
**Frequency (2/11)**			
	Fibromyalgia	“Aim for at least 30 minutes of aerobic exercise most days of the week.”	ACSM recommends an initial frequency of 1-2 days per week, gradually progressing to 2-3 days per week [1].
	Alzheimer disease	“Engage in moderate aerobic exercise such as brisk walking, cycling, or swimming for at least 30 minutes a day, five days a week.”	ACSM recommends a frequency of 3 days per week [1].
**Intensty (1/17)**			
	Hypertension	“Avoid high-intensity exercises: Avoid high-intensity exercises that can cause sudden increases in blood pressure, such as sprinting or heavy lifting.”	ACSM does not contraindicate vigorous-intensity aerobic exercise or heavy lifting assuming adequate progression, absence of underlying disease, and proper breathing technique (ie, avoidance of Valsalva maneuver) [1].
**Time (1/11)**			
	Fibromyalgia	“Start with 1-2 sets of 10-15 repetitions for each exercise and gradually increase the resistance as tolerated.”	ACSM recommends gradual progression of 4-5 to 8-12 repetitions and increasing from 1 to 2-4 sets per muscle group [1].
**Type (1/26)**			
	Hypertension	“Aim for at least 30 minutes of moderate-intensity aerobic exercise most days of the week.”	New ACSM guidelines reinforce that emphasis is no longer placed on aerobic exercise alone. Aerobic or resistance exercise alone or aerobic and resistance exercise combined (ie, concurrent exercise) is recommended on most, preferably all, days of the week to total 90 to 150 minutes per week or more of multimodal, moderate-intensity exercise [39].
**Volume (0/8)**			
	N/A^c^	N/A	N/A
**Progression (1/15)**			
	Overweight and obesity	“If you’re new to exercise, start with low-intensity activities such as walking or swimming, and gradually increase your intensity and duration.”	ACSM recommends initial intensity should be moderate, progressing to vigorous for greater health benefits [1].
**Special considerations (0/23)**			
	N/A	N/A	N/A
**References (0/2)**			
	N/A	N/A	N/A

^a^AI: artificial intelligence.

^b^ACSM: American College of Sports Medicine.

^c^N/A: not applicable.

### Readability Metrics

Average and individual readability metrics and word count for AI-generated exercise recommendations are provided in Table 3. On average, AI-generated output was 259.3 (SD 49.1) words (range 171-354) and considered “difficult to read” with an average Flesch reading ease of 31.1 (SD 7.7; range 14.5-47.3) and written at a college-level (mean 13.7, SD 1.7; range 10.1-18.0).

**Table 3:** Readability metrics for artificial intelligence–generated exercise recommendations by population.

Population	Word count	Flesch reading ease	Grade level
Healthy adults	187	14.5	15.2
Children and adolescents	253	29.8	14.1
Pregnancy	267	34.7	13.5
Older adults	276	37.0	12.2
Cardiovascular disease	271	33.6	13.2
Heart failure	235	23.0	16.2
Heart transplant	278	24.9	14.4
Peripheral artery disease	322	32.4	13.4
Cerebrovascular accident	346	22.0	15.1
Asthma	317	41.1	12.0
COPD^a^	247	47.3	10.1
Diabetes	201	36.7	11.8
Dyslipidemia	291	19.6	15.9
Hypertension	247	34.5	13.3
Overweight and obesity	200	34.7	13.2
Arthritis	236	38.4	13.0
Cancer	319	24.8	14.9
Fibromyalgia	303	40.0	12.2
HIV	232	30.0	13.9
Kidney disease	354	31.1	15.3
Multiple sclerosis	255	38.4	11.4
Osteoporosis	171	32.7	12.3
Spinal cord injury	281	25.5	14.1
Alzheimer disease	191	29.1	14.8
Intellectual disability	241	32.1	13.2
Parkinson disease	221	19.8	18.0
Mean (SD)	259.3 (49.1)	31.1 (7.7)	13.7 (1.7)

^a^COPD: chronic obstructive pulmonary disease.

### Qualitative Analysis

A secondary aim of this study was to identify potential patterns, consistencies, and gaps in AI-generated exercise recommendation text outputs. Major observations derived from qualitative evaluation of AI-generated exercise recommendations can be found in Multimedia Appendix 2. Briefly, several recurring themes emerged among the total sample, including liability and safety, preference for aerobic exercise, and inconsistencies in the terminology used for exercise professionals. Importantly, AI-generated output showed potential bias and discrimination against certain age-based populations and individuals with disabilities. The implications of these findings are discussed in detail below.

## Discussion

### Principal Findings

This study sought to explore the suitability of AI-generated exercise recommendations using a popular generative AI platform, ChatGPT. Given the recent launch and popularity of ChatGPT and other similar generative AI platforms, the overall goal was to formally appraise the suitability and readability of AI-generated output likely to be seen by patients and inform exercise and health care professionals and other stakeholders on the potential benefits and limitations of using AI to leverage for patient education. The major findings were that AI-generated output (1) presented 41.2% (107/260) of the content provided in a gold-standard exercise recommendation indicating poor comprehensiveness; (2) of the content provided, chat output was 90.7% (146/161) accurate with most discordance related to the need for exercise preparticipation health screening; and (3) had college-level readability.

The results of this study are consistent with a recently published research letter that evaluated the appropriateness of CVD prevention recommendations from ChatGPT [40]. Sarraju et al [40] developed 25 questions on fundamental heart disease concepts, posed them to the AI interface, and subjectively graded responses as “appropriate” or “inappropriate.” AI-generated responses were deemed to be 84% appropriate with noted misinformation provided for questions surrounding ideal exercise volume and type for health and heart disease prevention. This study expands upon these findings by focusing on ExRx, testing additional metrics (ie, comprehensiveness and readability) using an objective, formal coding system based on a ground truth source, and in an expanded list of clinical populations.

### Real-World Implications of These Findings

Our findings suggest that while AI-generated exercise recommendations are generally accurate (146/161, 90.7%), they may lack comprehensiveness in certain critical components of ExRx such as target frequency, intensity, time, and type of exercise, which could potentially hinder ease of implementation or their effectiveness. The most common (ie, 8/15, 53%) source of misinformation was the recommendation to seek medical clearance prior to engaging in any exercise. Potential downstream implications are undue patient concern and triggering an unnecessary number of adults for medical evaluation, both posing as potential barriers to exercise adoption [41,42].

The ACSM preparticipation screening guidelines emphasize the public health message that exercise is important for all individuals and that the preparticipation health screening should not be a deterrent to exercise participation [41]. The preparticipation screening algorithm considers current physical activity levels, desired exercise intensity, and the presence of known or underlying CVD, metabolic, and renal disease. Following this algorithm, lesser than 3% of the general population would be referred before beginning vigorous exercise, and approximately 54% would be referred before beginning any exercise [42]. Interestingly, exercise professionals are well-equipped to facilitate preparticipation screening, yet AI-generated output disproportionately emphasized medical clearance by a health care provider or doctor prior to working with an exercise professional. In reference to exercise professionals, ChatGPT used varying and incorrect terminology such as “licensed exercise physiologist” that does not reflect current-state credentialing for exercise professionals working with clinical populations (ie, ACSM Certified Clinical Exercise Physiologist [43]). These findings corroborate with existing challenges in the public health’s understanding of the role of exercise professionals, levels of qualification, and respective scope of practice [44].

As AI-based technologies continue to evolve, striking the right balance between medical precision and risk mitigation remains a crucial consideration [45]. The question of how definitive an AI-based model should be when delivering medical education is multifaceted. On the one hand, the inclination of the AI-based model toward vague or general recommendations can be seen as a responsible stance to mitigate risks. On the other hand, there is merit in AI-based models providing clear, specific, and contextual guidance that reinforces evidence-based recommendations. This approach ensures that end users receive accurate and tailored advice, which is important in the context of medical education. This tension highlights the need for continued dialogue on how AI can enhance health care while ensuring that recommendations align with the highest standards of accuracy and patient safety. These discussions will be instrumental in shaping the future of AI-augmented health care.

### AI-Generated Output Least Accurate for Populations With Hypertension

Interestingly, the hypertension exercise recommendations scored the poorest (ie, highest discordance) with 57% (4/7) accuracy and misinformation surrounding the need for medical clearance and the recommended intensity and type of exercise (Table 2). For example, AI-generated output recommended avoiding high-intensity exercise “such as sprinting or heavy lifting”; however, the ACSM does not contraindicate vigorous-intensity exercise considering comorbidities and assuming adequate progression and proper technique [1]. Additionally, AI-generated output recommended a target exercise goal of “30 minutes of moderate-intensity aerobic exercise most days of the week.” Notably, the ACSM guidelines reinforce that emphasis is no longer placed on aerobic exercise alone but rather recommend aerobic and resistance exercise alone or combined (ie, concurrent exercise) on most, preferably all, days of the week to total 90-150 minutes per week or more of multimodal, moderate-intensity exercise [39]. Reasons for this discordance are likely because the ChatGPT model relies on training data preceding 2021 and may not capture real-time research advancements. Nevertheless, these findings are important because hypertension is the most common, costly, and modifiable CVD risk factor with strong evidence-based and guideline-driven recommendations, whereby support of exercise is a critical component of first-line treatment for elevated blood pressure [7,46-48].

### Social Determinants of Health Considerations

Not surprisingly, our evaluation of this AI-based technology identified social determinants of health considerations regarding educational obtainment for its users. Average readability of the AI-generated output was found to be very high, at the college level, which poses significant challenges for the majority of patients, as The National Institutes of Health, American Medical Association, and American Heart Association all recommend that patient education materials be written at or below a sixth-grade reading level [49] based on national educational obtainment trends. Poor readability of patient materials can exacerbate disparities in access to care for those with limited health literacy, and those individuals may experience more barriers to understand and apply the information provided [29,30]. These findings highlight the need for ongoing evaluation and refinement of AI-generated educational output to prevent inappropriate recommendations that do not improve disparities in clinical outcomes. AI-based models, such as ChatGPT, and their output are vulnerable to both poor data quality and noninclusive design. Notably, AI-generated output used different tenses and pronouns depending on the demographic group being addressed, which potentially perpetuates digital discrimination including stereotypes and biases (Multimedia Appendix 2). For instance, most AI-generated exercise recommendations were provided in the second-person tense; however, recommendations for individuals with intellectual disabilities, older adults, and children and adolescents were written in the third-person tense with the AI-based model, assuming these populations were not the primary end users. Additionally, most exercise examples provided by the chatbot were activities favoring ambulating individuals (eg, walking and running) potentially limiting education for, and perpetuating bias against, individuals with disabilities. Generative AI can contribute to bias or discrimination in several ways, beginning with the use of biased data to train AI-based models that learn and perpetuate biases in its output [50]. Additionally, AI-based models may be designed with certain features that result in biased or discriminatory outputs, such as using certain variables that are correlated with gender or race [50]. Put in practice, AI-based models can further extend societal biases and stereotypes by relying on existing patterns and trends in the data that reinforce gender or racial stereotypes [50]. These findings highlight the need for caution in using generative AI for health education and the importance of careful consideration of potential biases and discriminatory language.

To summarize, this study demonstrates that AI-generated exercise recommendations hold some promise in accurately providing exercise information but are not without issues (ie, gaps in critical information, biases, and discrimination) that could lead to potentially harmful consequences. The art of ExRx involves considering individual factors and nuances that may not be fully captured by technology [1]. Factors such as medical history, medications, personal preferences, health and physical literacy, and physical limitations are just a few examples of the complexities involved in creating an individualized exercise plan [1]. It is important to note that AI-generated output often lacks references to primary sources or literature, underscoring the need for health care provider oversight in interpreting and verifying the validity of the information presented. In this study, the reference sources provided were 100% accurate (2 of 2); however, “hallucinations” of fabricated or inaccurate references are quite common and are a growing concern for AI-generated medical content [51].

### Limitations

There are limitations to this study. This evaluation was limited to a single generative AI platform, which may not be representative of all LLM programs. Additionally, this study is limited to a specific time period and topic, and the findings may not be generalizable to other topics or time periods. Importantly, this model was evaluated using a single, structured prompt that can potentially lead to overfitting or superficial outputs and compromise generalizability. The lack of exposure to a range of prompts makes it challenging to discern if outcomes truly reflect the model’s capabilities or are specific to the nature of the provided prompt. Given that LLMs can yield varied outcomes based on prompts, this limitation is critical for the interpretation and application of the model’s results across various scenarios. This approach was selected as it most closely recapitulates how a publicly available chatbot would likely be used in a real-world setting by an inexperienced end user (ie, lacking knowledge of prompt methodologies). Indeed, all (N=26) AI-generated exercise recommendations were coherent, contextual, and relevant suggesting that the standardized single prompt was structured to elicit an appropriate response. However, it is likely that additional prompt engineering considerations (ie, specificity, iteration, and roles and goals) will yield incremental capabilities and superior model performance than reported in this study. Future work should consider advanced and diverse prompts to assess the model’s robustness across various scenarios. The results rely on the accuracy of the coders in identifying relevant content and assessing its accuracy. The high level of agreement between raters suggests that the coding scheme was well-defined and easily interpretable; however, there is potential for observer bias due to the raters’ shared mentorship, research training, and educational experiences. It is also worth noting that this study used the Flesch-Kincaid formula to assess readability that has known limitations, such as not accounting for the complexity of ideas and vocabulary and not considering readers' cultural and linguistic backgrounds [36]. This tool was selected due to its objectivity, standardization, and the fact that scores are computationally derived, which lowers the risk of human error, thus rendering it the most appropriate tool to address this research question [36]. Nevertheless, future research may benefit from examining the Flesch-Kincaid formula in conjunction with other measures to gain a more comprehensive understanding of AI-generated output readability.

Despite the noted limitations, this study possesses several strengths. To the best of our knowledge, this study is the first to report on the quality of AI-generated exercise recommendations for individuals across the life span (ie, children and adolescents, healthy adults, and older adults) and for 23 additional clinical populations. A major strength of this study is the use of a formal grading framework with a double-coding system to objectively assess the comprehensiveness and accuracy of the AI-generated exercise recommendations, which extends the literature and increases the reliability and validity of these findings [40]. Adding to its credibility, this grading system was developed and refined by experts in the field of exercise science, including a former associate editor [35], editor, and contributing author [1] of the ACSM GETP (LSP and ALZ). Multiple measures were used to assess the suitability of AI-generated recommendations and its potential for digital discrimination. Recommendations were evaluated by their comprehensiveness, accuracy, and readability, which provided a thorough summarization of the strengths and weaknesses of AI-generated content. The output was compared to well-established evidence-based guidelines (ie, ACSM GETP) as a gold-standard reference, which strengthens the validity of the results. Finally, the standardization of queries in this study minimized bias and allowed for an objective evaluation of the AI-generated exercise recommendations. These structured prompts were integral to the research design, shaping the language model's responses and enabling the systematic evaluation of its performance against ACSM GETP as the ground truth benchmark. This methodological approach ensures that the outcomes presented in this study are grounded in a consistent and rigorously designed interaction process.

### Future Directions

Given the recent development of open-source generative AI technologies, this area is ripe for exploration. However, before proceeding with extensive randomized controlled trials, it is crucial to prioritize the safety and ethical considerations associated with AI-generated medical education. As AI technologies have the potential to impact health disparities, it is essential to carefully evaluate their use to ensure inclusivity and appropriate messaging across demographics [27,52-54]. Further research is needed to develop, test, and implement AI technologies that serve individuals safely, effectively, and ethically without perpetuating bias, discrimination, or causing harm. This includes exploring ways to mitigate potential biases and discriminatory outcomes. Outside of the research setting, health care and exercise professionals can play a crucial role in improving AI-based models through prompting and by giving corrective feedback to retrain biases and inaccuracies in AI-generated responses. By enriching ChatGPT with user-specific data including exercise components, literacy level, physical limitations, and other activity considerations, there are opportunities to improve the personalization of recommendations and lessen digital discrimination. Through this stewardship, continuous refinement will likely improve the performance, usability, and appropriateness of the model, translating to superior patient outcomes, which is the goal of provider-enablement and patient-facing tools. As LLMs continue to evolve, it will become increasingly important for researchers to continuously assess improvements with response variations over time. Importantly, future work should explore the incremental value of advanced and diverse prompting considerations. Examples of prompting considerations include the provision of roles and goals (eg, “You are a Clinical Exercise Physiologist and your goal is to design a safe and effective exercise prescription to lower blood pressure”), engaging in multiple or chain prompting and specifically prompting for content commonly missing from output as identified in this study.

To ensure the responsible and safe deployment of AI technologies in health care, conducting thorough implementation studies is a logical next step. These studies should focus on measuring various factors, including acceptability, adoption, appropriateness, costs, feasibility, fidelity, penetration, and sustainability. By thoroughly investigating these implementation aspects, we can ensure that the technology is well-integrated and does not pose any harm to patients or health care systems. Following the completion of the implementation studies, it is important to assess the impact of AI-generated models on service outcomes. This includes evaluating health care quality factors such as safety, timeliness, efficiency, effectiveness, equity, and patient-centeredness [55]. Understanding how AI technologies influence these service outcomes will provide valuable insights into their overall impact on health care delivery. Additionally, measuring patient-centered and end-user outcomes is essential to evaluate the effectiveness of AI technologies in improving patient experiences and outcomes. Randomized controlled trials designed to test ChatGPT as an intervention to augment behavior change and associated health outcomes would be of great public health interest. These trials should prioritize patient-centered outcomes, including satisfaction, usability, experience, and patient activation [56]. By assessing these outcomes, we can determine the effectiveness of AI technologies in empowering patients and fostering meaningful engagement with health care providers.

### Conclusions

To conclude, this study found that AI-generated exercise recommendations have moderate comprehensiveness and high accuracy when compared to a gold-standard reference source. However, there are notable gaps in content surrounding critical components of ExRx and potentially biased and discriminatory outputs. Additionally, the readability level of the recommendations may be too high for some patients, and the lack of references in AI-generated content may be a significant limitation for use. Health care providers and patients may wish to remain cautious in relying solely on AI-generated exercise recommendations and should limit their use in combination with clinical expertise and oversight.

## Appendix

Multimedia Appendix 1 Output from artificial intelligence–generated exercise recommendations for clinical populations (N=26).

Multimedia Appendix 2 Summary of major themes derived from artificial intelligence–generated exercise recommendations.

## Abbreviations

ACSM: American College of Sports Medicine
AI: artificial intelligence
CVD: cardiovascular disease
ExRx: exercise prescription
FITT: frequency, intensity, time, and type
GETP: Guidelines for Exercise Testing and Prescription
GPT: generative pretrained transformer
LLM: large language model
