# Supplementary material for: Comprehensiveness, Accuracy, and Readability of Exercise Recommendations Provided by an AI-Based Chatbot: Mixed Methods Study
Source: JMIR Med Educ. 2024 Jan 11;10:e51308. doi: 10.2196/51308 (PMC10811574; doi:10.2196/51308)
Supplement: Multimedia Appendix 1 [file mededu_v10i1e51308_app1.pdf]

## 1. Healthy adults

Here are some exercise recommendations for healthy adults:

Aerobic Exercise: The American Heart Association recommends at least 150 minutes of moderate-intensity aerobic exercise or 75 minutes of vigorous-intensity aerobic exercise per week. Examples of moderate-intensity aerobic exercise include brisk walking, cycling, and swimming. Vigorous-intensity aerobic exercise includes running, fast cycling, and high-intensity interval training (HIIT).

Strength Training: Strength training helps build muscle and increase bone density. It's recommended that adults do muscle-strengthening exercises at least two days a week. Examples of strength training exercises include weightlifting, resistance band workouts, and bodyweight exercises like push-ups, squats, and lunges.

Flexibility and Balance: Flexibility and balance exercises are important for maintaining range of motion and preventing falls. Yoga and Pilates are great options for improving flexibility and balance.

Active Lifestyle: In addition to structured exercise, it's important to maintain an active lifestyle by incorporating physical activity into your daily routine. This could include things like taking the stairs instead of the elevator, walking or biking to work, or doing household chores.

Remember to always listen to your body and consult with a healthcare professional before starting a new exercise routine.

## 2. Children and adolescents

Regular physical activity is important for children and adolescents to maintain good health and well-being. Here are some exercise recommendations for children and adolescents:

1. Age-appropriate activities: Children and adolescents should engage in activities that are appropriate for their age and developmental stage. For example, preschoolers may enjoy activities such as running, jumping, and playing with balls, while older children may be interested in sports or structured activities such as dance classes or martial arts.

2. Moderate to vigorous exercise: Children and adolescents should engage in moderate to vigorous physical activity for at least 60 minutes per day. This can include activities such as running, swimming, dancing, cycling, or team sports.

3. Strength training: Strength training can be incorporated into a child's exercise routine, but should be supervised by an adult and focused on bodyweight exercises or resistance bands.

4. Fun and variety: Exercise should be fun and varied to keep children and adolescents engaged and motivated. Encourage them to try new activities and explore different types of exercise.

5. Limit screen time: Children and adolescents should limit sedentary screen time, including television, video games, and computers, to no more than 2 hours per day.

6. Safety first: Safety is paramount when it comes to exercise for children and adolescents. Make sure they wear appropriate clothing and footwear, use protective equipment when necessary, and are supervised when engaging in activities such as swimming or cycling.

Remember, children and adolescents learn by example, so encourage and model healthy exercise habits for them to follow.

## 3. Pregnancy

Exercise can have many benefits during pregnancy, such as helping to improve cardiovascular health, reduce stress, improve mood, and even help prepare for childbirth. However, it is important to consult with your healthcare provider before starting or continuing an exercise program during pregnancy, especially if you have any medical conditions or complications.

Assuming your healthcare provider has given you the green light to exercise during pregnancy, here are some general recommendations:

1. Low-impact aerobic exercises: Walking, swimming, stationary cycling, and low-impact aerobics are great options for pregnant women because they are easy on the joints and do not put too much strain on the body.
2. Strength training: Weight training with light weights or resistance bands can help maintain muscle strength and tone, which can be helpful during pregnancy and postpartum.
3. Pelvic floor exercises: The pelvic floor muscles support the bladder, uterus, and rectum. Strengthening these muscles can help prevent incontinence during and after pregnancy. Kegels exercises are a common type of pelvic floor exercise that involves contracting and relaxing the muscles in the pelvic floor.
4. Yoga or Pilates: These exercises can help improve flexibility and balance, as well as reduce stress and anxiety. However, it is important to avoid poses that involve lying on the back or twisting the abdomen.
5. Sports and activities to avoid: Contact sports, high-impact activities, and activities with a risk of falling, such as skiing or horseback riding, should be avoided during pregnancy.

Remember to listen to your body and make adjustments as needed. Stop exercising and consult your healthcare provider if you experience any unusual symptoms, such as vaginal bleeding, contractions, or dizziness.

## 4. Older adults

Regular exercise is important for people of all ages, including older adults. Exercise can help improve strength, flexibility, balance, and overall health, while also reducing the risk of chronic diseases such as heart disease, diabetes, and osteoporosis. However, it's important for older adults to choose exercises that are appropriate for their age and fitness level, and to check with their doctor before starting a new exercise program.

Here are some exercise recommendations for older adults:

Aerobic exercise: Aerobic exercise, also known as cardio, is any exercise that gets your heart rate up and increases your breathing. Good examples for older adults include walking, swimming, cycling, or low-impact aerobics. Aim for at least 150 minutes of moderate-intensity aerobic exercise per week.

Strength training: Strength training can help maintain muscle mass and bone density, which is particularly important as we age. Resistance bands, dumbbells, or weight machines can be used for strength training. Aim to do strength training exercises for all major muscle groups at least twice a week.

Flexibility and balance exercises: Yoga, stretching, and tai chi are all good examples of exercises that can improve flexibility and balance. These types of exercises can help reduce the risk of falls and injuries. Aim to do these exercises at least twice a week.

Functional exercises: Functional exercises mimic everyday movements and can help improve the ability to perform activities of daily living. Examples of functional exercises include squats, lunges, and step-ups.

Remember to start slowly and gradually increase the intensity and duration of your exercise routine. It's also important to stay hydrated, wear appropriate footwear and clothing, and listen to your body for signs of pain or discomfort.

## 5. Cardiovascular disease

Exercise is an important part of the treatment and management of cardiovascular disease. Regular physical activity can help to improve heart health, lower blood pressure, reduce the risk of heart attack and stroke, and improve overall cardiovascular function.

Here are some exercise recommendations for cardiovascular disease:

1. Start Slowly: If you have been inactive for a while, it's important to start slowly and gradually increase your activity level. You can begin with short, low-intensity workouts and gradually increase the duration and intensity of your workouts over time.
2. Aim for 150 Minutes of Moderate-Intensity Exercise Per Week: The American Heart Association recommends at least 150 minutes of moderate-intensity exercise per week for cardiovascular health. This can include activities such as brisk walking, cycling, swimming, or dancing.
3. Strength Training: Resistance training can also be beneficial for cardiovascular health. It can help to improve muscle strength, which can in turn improve cardiovascular function. Aim for at least two days per week of strength training exercises that target all major muscle groups.

4. Consult with Your Doctor: It's important to consult with your doctor before beginning any exercise program, especially if you have a history of cardiovascular disease or other medical conditions.
5. Make It a Habit: Consistency is key when it comes to exercise and cardiovascular health. Make exercise a regular part of your routine and aim for daily activity, even if it's just a short walk or some light stretching.
6. Listen to Your Body: It's important to listen to your body and adjust your exercise routine as needed. If you experience chest pain, dizziness, or shortness of breath during exercise, stop immediately and seek medical attention.

## 6. Heart failure

Exercise is an important component of managing heart failure, as it can help improve cardiovascular function, decrease symptoms, and enhance quality of life. However, it is important to consult with a healthcare professional before starting any exercise program, as the type and intensity of exercise may need to be tailored to the individual's specific needs and limitations.

Here are some general exercise recommendations for heart failure:

Start slowly and gradually increase the duration and intensity of exercise over time.

Focus on low-impact activities, such as walking, cycling, swimming, or light resistance training, which can improve cardiovascular fitness without putting too much stress on the joints.

Aim for at least 30 minutes of moderate-intensity exercise most days of the week, or as recommended by your healthcare provider.

Consider incorporating interval training, which involves alternating periods of high-intensity exercise with periods of rest or low-intensity exercise, as it has been shown to be effective in improving cardiovascular function in heart failure patients.

Be sure to warm up before exercise and cool down afterwards to help prevent injury and minimize symptoms.

Pay attention to your body and symptoms, and stop exercising if you experience chest pain, shortness of breath, dizziness, or any other concerning symptoms.

Remember, it's important to work with your healthcare provider to develop an exercise program that is safe and appropriate for you based on your specific medical condition, fitness level, and other individual factors.

## 7. Heart transplant

It's important to note that exercise recommendations for heart transplant recipients should be individualized and based on the individual's overall health, level of physical activity, and any specific limitations or recommendations from their healthcare provider. That being said, here are some general guidelines for exercise after a heart transplant:

Start slowly: It's important to start with low-intensity exercises and gradually increase the intensity and duration over time. This will help to prevent injury and allow your body to adjust to the new demands being placed on it.

Focus on aerobic exercise: Aerobic exercise is beneficial for cardiovascular health and can help to improve endurance and stamina. Examples of aerobic exercise include brisk walking, cycling, swimming, or using a stationary bike or elliptical machine.

Incorporate resistance training: Resistance training can help to build muscle and improve overall strength. Examples of resistance training exercises include weightlifting, resistance band exercises, and bodyweight exercises such as push-ups and squats.

Avoid high-impact activities: High-impact activities such as running and jumping can put stress on the joints and may not be appropriate for all heart transplant recipients. Talk to your healthcare provider about what activities are safe for you.

Listen to your body: Pay attention to any signs of fatigue, dizziness, or discomfort during exercise. If you experience any of these symptoms, stop exercising and talk to your healthcare provider.

Follow your healthcare provider's recommendations: Your healthcare provider may have specific recommendations or restrictions for exercise based on your individual health status. It's important to follow these recommendations to ensure your safety and optimal health.

Remember to always consult with your healthcare provider before starting any new exercise program, especially after a heart transplant.

## 8. PAD

Peripheral arterial disease (PAD) is a condition that results from narrowed or blocked arteries, usually in the legs, which can cause pain, cramping, and weakness during physical activity. Exercise is an important part of managing PAD, and here are some general exercise recommendations:

Consult with your healthcare provider: Before starting any exercise program, it's important to talk to your healthcare provider, especially if you have PAD. They can help you develop a safe and effective exercise plan that takes into account your specific needs and limitations.

Start with low-intensity exercise: Begin with low-intensity exercises such as walking on a flat surface or cycling at a slow pace. Gradually increase the duration and intensity of your exercise sessions over time.

Incorporate aerobic exercise: Aerobic exercise is important for improving circulation and can help to reduce symptoms of PAD. Examples of aerobic exercise include walking, cycling, swimming, and using a stationary bike or elliptical machine.

Consider interval training: Interval training involves alternating short bursts of high-intensity exercise with periods of rest or low-intensity exercise. This type of training has been shown to be effective for improving cardiovascular health in people with PAD.

Include strength training: Strength training exercises can help to improve muscle strength and endurance, which can make it easier to perform daily activities. Examples of strength training exercises include weightlifting, resistance band exercises, and bodyweight exercises such as squats and lunges.

Avoid high-impact activities: High-impact activities such as running and jumping can put stress on the joints and may not be appropriate for all people with PAD. Talk to your healthcare provider about what activities are safe for you.

Wear appropriate footwear: Wear supportive, comfortable shoes that fit well and provide good arch support to reduce the risk of foot injuries.

Remember to listen to your body and avoid pushing yourself too hard during exercise. If you experience any pain, discomfort, or other symptoms, stop exercising and talk to your healthcare provider.

## 9. Cerebrovascular accident

Cerebrovascular accident (CVA), also known as a stroke, can cause a range of physical and cognitive impairments that may impact one's ability to engage in physical activity. However, exercise can be an important part of rehabilitation after a stroke. Here are some general exercise recommendations:

Consult with your healthcare provider: Before starting any exercise program after a stroke, it's important to talk to your healthcare provider, especially if you have any physical or cognitive limitations. They can help you develop a safe and effective exercise plan that takes into account your specific needs and limitations.

Start with low-intensity exercise: Begin with low-intensity exercises such as stretching or range-of-motion exercises to help improve flexibility and reduce muscle stiffness. Gradually increase the duration and intensity of your exercise sessions over time.

Include aerobic exercise: Aerobic exercise can help to improve cardiovascular health, endurance, and overall physical function after a stroke. Examples of aerobic exercise include walking, cycling, swimming, and using a stationary bike or elliptical machine.

Incorporate strength training: Strength training exercises can help to improve muscle strength and endurance, which can make it easier to perform daily activities. Examples of strength training exercises include weightlifting, resistance band exercises, and bodyweight exercises such as squats and lunges.

Focus on balance and coordination: After a stroke, many people may experience balance and coordination difficulties. Exercises that focus on improving balance and coordination can help to reduce the risk of falls and improve overall physical function. Examples of balance exercises include standing on one leg, walking heel-to-toe, and standing on a wobble board.

Include cognitive exercises: Cognitive exercises, such as puzzles, memory games, and problem-solving activities, can help to improve cognitive function after a stroke.

Consider aquatic therapy: Aquatic therapy can be a good option for people with mobility or balance issues as it provides a low-impact environment that can support the body and reduce the risk of injury.

Remember to listen to your body and avoid pushing yourself too hard during exercise. If you experience any pain, discomfort, or other symptoms, stop exercising and talk to your healthcare provider.

## 10. Asthma

Asthma is a chronic respiratory condition that can be triggered by physical activity or exercise. However, exercise can still be an important part of managing asthma and improving overall respiratory health. Here are some general exercise recommendations:

Consult with your healthcare provider: Before starting any exercise program, it's important to talk to your healthcare provider, especially if you have asthma. They can help you develop a safe and effective exercise plan that takes into account your specific needs and limitations.

Warm-up: A proper warm-up can help to prepare the body for exercise and reduce the risk of asthma symptoms. Try walking or cycling at a low intensity for 5-10 minutes before beginning your workout.

Choose low-impact activities: Low-impact activities such as walking, swimming, cycling, and using an elliptical machine or rowing machine can be good options for people with asthma. These activities typically involve less intense breathing and less impact on the joints.

Consider interval training: Interval training involves alternating short bursts of high-intensity exercise with periods of rest or low-intensity exercise. This type of training has been shown to be effective for improving cardiovascular health in people with asthma.

Monitor your breathing: Pay attention to your breathing during exercise and take breaks if you begin to feel short of breath. Try to breathe through your nose as much as possible, as this can help to filter and humidify the air.

Carry your inhaler: Make sure to have your inhaler with you during exercise in case of an asthma flare-up. Use it as prescribed by your healthcare provider.

Exercise indoors on high pollen days: If you are sensitive to pollen, try to exercise indoors on high pollen days to reduce the risk of triggering asthma symptoms.

Remember to listen to your body and avoid pushing yourself too hard during exercise. If you experience any pain, discomfort, or other symptoms, stop exercising and talk to your healthcare provider.

## 11. COPD

Exercise can be beneficial for people with chronic obstructive pulmonary disease (COPD) by improving their breathing and overall fitness. However, it is important to work with a healthcare professional to develop an exercise plan that is safe and effective for you. Here are some general guidelines and examples of exercises that may be beneficial for people with COPD:

1. Start slow and gradually increase intensity and duration of exercise over time.
2. Warm up before exercise by doing some light stretching or low-intensity exercise.
3. Incorporate both aerobic and strength training exercises into your routine.
4. Use pursed-lip breathing during exercise to help regulate your breathing and decrease shortness of breath.
5. Stay hydrated and take breaks as needed.

Examples of exercises for people with COPD:

1. Walking: This is a low-impact aerobic exercise that can be done indoors or outdoors. Start with short walks and gradually increase the duration and distance.
2. Cycling: Cycling can be done on a stationary bike or outdoors. It is a good low-impact aerobic exercise that can be done at your own pace.
3. Resistance training: This type of exercise can help build muscle strength and improve overall fitness. Use light weights or resistance bands and start with a few repetitions before gradually increasing.
4. Yoga: Yoga can help improve breathing, flexibility, and overall fitness. Look for classes or videos that are specifically designed for people with COPD.

Remember, it is important to work with a healthcare professional to develop an exercise plan that is safe and effective for you.

## 12. Diabetes

Exercise is an essential component of diabetes management, and it can help improve blood sugar control, increase insulin sensitivity, and reduce the risk of complications associated with diabetes. Here are some exercise recommendations for people with diabetes:

1. Aim for at least 150 minutes of moderate-intensity aerobic exercise per week spread out over at least three days. Examples of moderate-intensity exercise include brisk walking, cycling, or swimming.
2. Include resistance training in your exercise routine at least twice a week. Resistance training helps build muscle, which can improve insulin sensitivity.

3. Stay active throughout the day. Try to break up long periods of sitting with short bursts of physical activity, such as walking or stretching.
4. Choose activities that you enjoy and that fit your lifestyle. This will make it easier to stick with your exercise routine.
5. Monitor your blood sugar before and after exercise to ensure it stays within a safe range.
6. If you have complications related to diabetes, such as neuropathy or retinopathy, consult with your healthcare provider before starting any new exercise program.

Remember to start slowly and gradually increase the intensity and duration of your exercise routine. It is also important to stay hydrated and wear comfortable, well-fitting shoes when exercising.

## 13. Dyslipidemia

Dyslipidemia refers to abnormal levels of lipids (such as cholesterol and triglycerides) in the blood, which can increase the risk of developing heart disease and other cardiovascular conditions. Exercise can play an important role in managing dyslipidemia. Here are some exercise recommendations for dyslipidemia:

1. Aerobic exercise: Aerobic exercise, such as brisk walking, cycling, or swimming, can help lower total cholesterol and triglyceride levels and increase levels of high-density lipoprotein (HDL) or "good" cholesterol. Aim for at least 150 minutes of moderate-intensity aerobic exercise per week, or 75 minutes of vigorous-intensity aerobic exercise per week.
2. Resistance training: Resistance training, such as weight lifting or bodyweight exercises, can help increase muscle mass and improve insulin sensitivity, which can lower triglyceride levels. Aim for at least two days per week of resistance training, targeting all major muscle groups.
3. High-intensity interval training (HIIT): HIIT involves short bursts of intense exercise followed by periods of rest or recovery. HIIT has been shown to improve lipid profiles in people with dyslipidemia. However, it should be approached with caution, especially for people who are not accustomed to high-intensity exercise or who have other health conditions.
4. Flexibility and balance exercises: Flexibility and balance exercises, such as yoga or tai chi, can improve overall fitness and reduce the risk of falls and other injuries. These types of exercises may not directly affect lipid levels but can still be beneficial for overall health.
5. Lifestyle modifications: In addition to exercise, lifestyle modifications such as a healthy diet, weight management, and quitting smoking can also help improve lipid profiles and reduce the risk of cardiovascular disease.

It's important to consult with a healthcare professional before starting a new exercise program, especially if you have a history of heart disease or other health conditions.

## 14. Hypertension

Regular exercise can be an effective way to lower blood pressure and manage hypertension. Here are some exercise recommendations for individuals with hypertension:

1. Consult with your doctor: Before starting any exercise program, it is important to consult with your doctor to make sure it is safe for you to exercise and to get guidance on the most appropriate type and intensity of exercise.
2. Aim for moderate-intensity exercise: Aim for at least 30 minutes of moderate-intensity aerobic exercise most days of the week. Examples of moderate-intensity activities include brisk walking, cycling, swimming, or dancing.
3. Incorporate strength training: Strength training exercises, such as lifting weights or using resistance bands, can also be beneficial for managing hypertension. Aim for two to three strength training sessions per week, targeting major muscle groups.
4. Avoid high-intensity exercises: Avoid high-intensity exercises that can cause sudden increases in blood pressure, such as sprinting or heavy lifting.
5. Warm up and cool down: Begin and end each exercise session with a few minutes of gentle stretching or low-intensity exercise to warm up and cool down.
6. Monitor your blood pressure: Keep track of your blood pressure before, during, and after exercise to ensure it remains within a safe range.
7. Be consistent: Consistency is key to reaping the benefits of exercise for hypertension management. Aim to exercise regularly and make it a part of your daily routine.

Remember to always listen to your body, start slowly and gradually increase the intensity and duration of your exercise routine over time.

**Note that guidelines were compared to new guidelines by Pescatello et al, available here**

## 15. Overweight and obesity

For overweight and obesity, it's important to engage in regular physical activity as part of an overall healthy lifestyle. Here are some exercise recommendations:

1. Start slow: If you're new to exercise, start with low-intensity activities such as walking or swimming, and gradually increase your intensity and duration.
2. Aim for at least 150 minutes of moderate-intensity aerobic exercise per week: This can be broken down into 30 minutes of exercise on most days of the week.

3. Incorporate strength training: Strength training can help build muscle mass and improve overall body composition. Aim for at least two days per week of strength training exercises targeting major muscle groups.
4. Mix it up: Variety is key to keeping exercise interesting and challenging. Incorporate a mix of aerobic and strength training exercises, and try new activities to keep your workouts fresh.
5. Seek professional guidance: If you're unsure about how to get started or what types of exercises are safe and effective for your current fitness level, consider working with a certified personal trainer or exercise specialist.
6. Consider lifestyle changes: Exercise alone may not be enough to achieve significant weight loss. Combining exercise with a healthy diet and other lifestyle changes can lead to better results.

## 16. Arthritis

Exercise can be very beneficial for people with arthritis, as it can help reduce pain, increase range of motion, and improve overall health and well-being. However, it's important to choose exercises that are safe and appropriate for your individual condition. Here are some general exercise recommendations for arthritis:

1. Low-impact aerobic exercises: These include activities such as walking, cycling, swimming, or water aerobics. These exercises can help improve cardiovascular health and endurance, while also being gentle on the joints.
2. Strength training: Strength training can help improve muscle strength and joint stability, which can help reduce the risk of falls and injury. However, it's important to use light weights and perform exercises that don't put too much stress on the joints.
3. Flexibility exercises: These include stretching and range-of-motion exercises, which can help improve flexibility and reduce stiffness in the joints.
4. Balance exercises: These exercises can help improve balance and reduce the risk of falls, which can be a concern for people with arthritis.

It's also important to listen to your body and adjust your exercise routine as needed. If you experience pain or discomfort during exercise, you may need to modify your routine or try a different type of exercise. Be sure to talk to your doctor or a physical therapist before starting any new exercise program, especially if you have a severe form of arthritis or other medical conditions that may affect your ability to exercise safely.

## 17. Cancer survivors

Exercise is an important part of cancer survivorship as it can help improve physical and emotional well-being, reduce the risk of cancer recurrence, and manage side effects of cancer treatment. However, the specific exercise recommendations for cancer survivors may vary depending on individual factors such as cancer type, treatment history, and current health status. It is always recommended to consult with a healthcare provider before starting or modifying an exercise routine.

Here are some general exercise recommendations for cancer survivors:

1. Start slowly and gradually increase intensity: It is important to start with low-intensity exercises and gradually increase the intensity over time. This can help prevent injury and ensure that the body is able to handle the exercise.
2. Choose a variety of exercises: Including a variety of exercises can help target different muscle groups and prevent boredom. Examples of exercises that may be beneficial for cancer survivors include walking, swimming, cycling, yoga, and resistance training.
3. Incorporate both aerobic and strength training exercises: Aerobic exercises such as walking, jogging, and cycling can help improve cardiovascular health and reduce the risk of cancer recurrence. Strength training exercises such as weightlifting and resistance bands can help improve muscle strength and bone density.
4. Listen to your body: Cancer survivors may experience fatigue, pain, or other side effects of treatment. It is important to listen to your body and adjust your exercise routine accordingly. Resting when needed and modifying exercises can help prevent injury and ensure that the body is able to recover properly.
5. Stay hydrated and well-nourished: Adequate hydration and nutrition are important for overall health and can help improve exercise performance. Drinking water before, during, and after exercise can help prevent dehydration, and consuming a balanced diet can provide the nutrients needed for energy and muscle recovery.

Remember to always consult with a healthcare provider before starting or modifying an exercise routine, especially if you have any medical conditions or are taking medications.

## 18. Fibromyalgia

Fibromyalgia is a chronic condition that causes widespread pain, fatigue, and tenderness in muscles and joints. Exercise can be an effective way to manage the symptoms of fibromyalgia, but it's important to choose the right type of exercise and to start slowly to avoid overexertion. Here are some exercise recommendations for fibromyalgia:

1. Low-impact aerobic exercise: Activities such as walking, cycling, and swimming can improve cardiovascular fitness, reduce pain, and improve overall mood. Start with short durations (10-15 minutes) and gradually increase the time as tolerated. Aim for at least 30 minutes of aerobic exercise most days of the week.

2. Stretching: Gentle stretching exercises can help reduce muscle stiffness and improve flexibility. Yoga and Pilates are great options, but be sure to modify poses as necessary to avoid pain or discomfort.
3. Strength training: Resistance exercises with light weights or resistance bands can help improve muscle strength, reduce pain, and improve overall function. Start with 1-2 sets of 10-15 repetitions for each exercise and gradually increase the resistance as tolerated.
4. Tai Chi: Tai Chi is a gentle form of exercise that involves slow, flowing movements and deep breathing. It has been shown to improve balance, reduce pain, and improve overall quality of life in people with fibromyalgia.
5. Water exercise: Water aerobics or swimming can be a great low-impact option for people with fibromyalgia. The buoyancy of the water can help reduce joint stress and pain, while the resistance of the water can provide a gentle strength training workout.

It's important to work with a healthcare provider or a qualified exercise professional to develop a safe and effective exercise program that meets your individual needs and abilities. Start slowly and gradually increase the intensity and duration of your workouts as tolerated. Don't push through pain or fatigue, and be sure to rest and recover between workouts.

## 19. HIV

Regular exercise can be beneficial for people living with HIV. It can help improve cardiovascular health, reduce stress and anxiety, boost immune function, and enhance overall well-being. However, it's important to consult with a healthcare provider before starting any new exercise program, especially if you have advanced HIV or other health conditions.

Assuming your healthcare provider has cleared you for exercise, here are some general exercise recommendations:

1. Start slowly: If you are new to exercise, start with low-intensity activities such as walking or gentle yoga, and gradually increase the intensity and duration over time.
2. Incorporate a variety of exercises: Include a mix of aerobic exercise, such as brisk walking or cycling, and strength training, such as weight lifting or resistance bands.
3. Aim for at least 150 minutes of moderate-intensity exercise per week: This can be broken up into smaller sessions throughout the day.
4. Listen to your body: If you experience any pain, fatigue, or other symptoms, stop exercising and consult with your healthcare provider.
5. Stay hydrated: Drink plenty of water before, during, and after exercise to prevent dehydration.
6. Consider working with a certified exercise professional: They can help design an individualized exercise program based on your specific needs and abilities.

Remember, exercise is just one component of a healthy lifestyle. It's important to also prioritize a balanced diet, adequate sleep, stress management, and taking any medications as prescribed by your healthcare provider.

## 20. Kidney disease

When it comes to exercise recommendations for kidney disease, it's important to keep in mind that everyone's situation is unique, and that exercise recommendations may vary based on the severity and stage of the kidney disease, as well as any other health conditions that may be present.

That being said, here are some general guidelines and recommendations for exercising with kidney disease:

1. Consult with a healthcare provider: Before beginning any exercise program, it's important to consult with a healthcare provider, especially if you have kidney disease. Your doctor or a licensed exercise physiologist can help you design an appropriate exercise program based on your health status and personal needs.
2. Focus on low-impact exercises: Low-impact exercises such as walking, cycling, swimming, and water aerobics can be good options for people with kidney disease, as they are gentle on the joints and can help improve cardiovascular health without putting excess strain on the kidneys.
3. Monitor fluid intake: People with kidney disease often need to limit their fluid intake, so it's important to monitor your fluid intake before, during, and after exercise to prevent dehydration.
4. Avoid activities that may cause injury: High-impact activities such as running, jumping, and contact sports may increase the risk of injury, which can be especially problematic for people with kidney disease who may have compromised bone density or muscle strength. Consider lower-impact activities that reduce the risk of injury.
5. Build strength with resistance training: Resistance training such as weightlifting or resistance band exercises can help build muscle and improve bone density, both of which can be beneficial for people with kidney disease. However, it's important to use proper form and start with light weights or resistance bands to prevent injury.
6. Adjust exercise intensity as needed: Depending on your health status, you may need to adjust the intensity of your exercise program to prevent overexertion. Listen to your body and make adjustments as needed.

Overall, exercise can be an important part of a healthy lifestyle for people with kidney disease. However, it's important to work with your healthcare provider to design an appropriate exercise program based on your individual needs and health status.

## 21. MS

Exercise can be beneficial for people with multiple sclerosis (MS) as it can improve mobility, balance, strength, and overall fitness. However, the type and intensity of exercise may need to be modified based on the individual's symptoms and limitations. It is always recommended to consult with a healthcare professional before starting any exercise program.

Here are some exercise recommendations for people with MS:

1. Aerobic exercise: This type of exercise can help improve cardiovascular health and endurance. Examples include walking, swimming, cycling, and using an elliptical machine.
2. Strength training: Building and maintaining muscle strength can help improve balance and mobility. Resistance bands or weights can be used for exercises such as squats, lunges, and arm curls.
3. Stretching: Gentle stretching can help improve flexibility and reduce muscle tightness. Yoga or Pilates can be good options for stretching exercises.
4. Balance training: Balance exercises can help reduce the risk of falls and improve stability. Examples include standing on one leg, heel-to-toe walking, and using a balance board.
5. Tai chi: This low-impact exercise combines movements with deep breathing and relaxation techniques. It has been shown to improve balance, flexibility, and overall quality of life for people with MS.
6. Water exercises: Water exercises can be especially beneficial for people with MS as they reduce the impact on joints and provide resistance for strength training. Swimming or water aerobics can be good options.

Remember to start slowly and gradually increase intensity and duration as tolerated. It's important to listen to your body and stop or modify exercises that cause pain or fatigue.

## 22. Osteoporosis

Exercise can be a key component in managing osteoporosis. The following are some exercise recommendations for individuals with osteoporosis:

1. Weight-bearing exercises: These exercises involve bearing your own body weight and can help increase bone density. Examples include walking, hiking, dancing, stair climbing, and jogging.
2. Resistance exercises: These exercises involve using weights or resistance bands to strengthen muscles and bones. Examples include weightlifting, push-ups, squats, and lunges.
3. Balance exercises: These exercises can help prevent falls, which can lead to fractures. Examples include standing on one leg, heel-to-toe walking, and tai chi.

4. Low-impact exercises: These exercises can be easier on the joints and may be appropriate for those who have difficulty with weight-bearing exercises. Examples include swimming, cycling, and using an elliptical machine.

It's important to talk to your doctor or a qualified exercise professional before starting any new exercise program, especially if you have osteoporosis or other medical conditions. They can help you determine which exercises are safe and appropriate for you, and can provide guidance on how to perform them correctly.

## 23. Spinal cord injury

Exercise is an essential component of a healthy lifestyle for individuals with spinal cord injuries (SCI). The specific exercises and intensity will depend on the level of injury and individual capabilities. It is recommended that individuals with SCI consult with a healthcare professional, such as a physical therapist, before starting an exercise program.

Here are some general exercise recommendations for individuals with SCI:

1. Aerobic exercise: Aerobic exercise can improve cardiovascular health, increase endurance, and reduce the risk of obesity and cardiovascular disease. Wheelchair aerobics, hand cycling, and swimming are examples of aerobic exercises that can be adapted for individuals with SCI.

2. Resistance training: Resistance training can help build muscle strength and maintain bone density. Resistance bands, weight machines, and free weights can be used for resistance training.

3. Range of motion exercises: Range of motion exercises can help maintain flexibility and reduce the risk of contractures, which occur when muscles and joints become stiff and immobile. Passive range of motion exercises, where a caregiver or therapist moves the limbs through their full range of motion, can be beneficial for individuals with limited mobility.

4. Core strengthening exercises: Core strengthening exercises can help improve trunk stability and reduce the risk of falls. Examples include seated crunches, seated twists, and seated planks.

5. Flexibility exercises: Flexibility exercises can help maintain range of motion and reduce the risk of injury. Stretching exercises can be adapted for individuals with SCI, including seated hamstring stretches, seated quad stretches, and seated hip flexor stretches.

It is important to remember that exercise should be tailored to the individual's abilities and goals. Starting slowly and gradually increasing intensity and duration can help prevent injury and promote long-term adherence to an exercise program.

## 24. Alzheimers

Regular exercise can be beneficial for people with Alzheimer's disease, as it can improve their cognitive function, mood, and overall quality of life. Here are some exercise recommendations for people with Alzheimer's disease:

1. Aerobic Exercise: Engage in moderate aerobic exercise such as brisk walking, cycling, or swimming for at least 30 minutes a day, five days a week. This can help improve cardiovascular health and reduce the risk of developing other health conditions.
2. Strength Training: Incorporate strength training exercises such as weight lifting or resistance band exercises at least twice a week to help maintain muscle mass and strength.
3. Balance and Coordination Exercises: Practice balance and coordination exercises such as Tai Chi or yoga to help improve stability and reduce the risk of falls.
4. Mind-Body Exercises: Mind-body exercises such as meditation or deep breathing can help reduce stress and improve mood.
5. Social Activities: Participating in social activities such as group fitness classes or walking groups can help improve social connections and reduce feelings of isolation and loneliness.

It's important to work with a healthcare professional to develop a personalized exercise plan that takes into account the individual's health status and abilities.

## 25. Intellectual disability

When it comes to exercise recommendations for individuals with intellectual disabilities, it's important to keep in mind that everyone is different and has varying levels of abilities and needs. However, here are some general guidelines to consider:

1. Consult with a healthcare provider: Before starting any exercise program, it's important to consult with a healthcare provider to ensure that the program is safe and appropriate for the individual's needs and abilities.
2. Focus on low-impact activities: Low-impact exercises are typically safer and easier on the joints. Examples include walking, swimming, cycling, and yoga.
3. Incorporate strength training: Strength training can help improve muscle tone, balance, and overall strength. Simple exercises like squats, lunges, and push-ups can be effective.
4. Make it fun: It's important to make exercise fun and enjoyable for individuals with intellectual disabilities. Incorporate games, music, and other fun activities to keep them engaged.
5. Consider group activities: Group activities can be a great way to encourage socialization and increase motivation. Look for local community centers or fitness programs that cater specifically to individuals with intellectual disabilities.
6. Keep it consistent: Consistency is key when it comes to seeing results from exercise. Try to establish a regular exercise routine and stick to it as much as possible.

Overall, the most important thing is to tailor the exercise program to the individual's needs and abilities, and to make it fun and enjoyable so that they are more likely to stick with it over the long term.

## 26. Parkinsons disease

Exercise can be very beneficial for people with Parkinson's disease, as it can help to improve balance, flexibility, mobility, and overall quality of life. Here are some exercise recommendations for Parkinson's disease:

1. Aerobic exercise: Cardiovascular exercise, such as walking, cycling, or swimming, can help to improve heart health and reduce the risk of other health problems associated with Parkinson's disease, such as high blood pressure and diabetes.
2. Strength training: Resistance exercises, such as weight lifting or resistance band exercises, can help to improve muscle strength and reduce the risk of falls.
3. Balance and stability training: Exercises that focus on balance, such as standing on one foot or practicing Tai Chi, can help to improve balance and stability, which can be particularly beneficial for people with Parkinson's disease who are at risk of falling.
4. Stretching and flexibility exercises: Gentle stretching exercises can help to improve flexibility and reduce muscle stiffness and rigidity.
5. Dance therapy: Some studies have suggested that dance therapy, such as tango or ballroom dancing, can be particularly beneficial for people with Parkinson's disease, as it can help to improve balance, coordination, and overall mobility.

It's important to note that exercise recommendations should be tailored to each individual's needs and abilities, and should be done under the guidance of a healthcare professional, such as a physical therapist or exercise physiologist.
