# Supplementary material for: Comprehensiveness, Accuracy, and Readability of Exercise Recommendations Provided by an AI-Based Chatbot: Mixed Methods Study
Source: JMIR Med Educ. 2024 Jan 11;10:e51308. doi: 10.2196/51308 (PMC10811574; doi:10.2196/51308)
Supplement: Multimedia Appendix 2 [file mededu_v10i1e51308_app2.pdf]

Theme 1: Concern with liability: Every single recommendation (except for children and adolescents) recommended checking in with a healthcare professional (n=56 mentions) to support exercise initiation or seek medical clearance before starting any new exercise program.

Theme 2: Concern with safety / injury: There was a strong emphasis of the importance of "low-impact" exercise (n=28 mentions). For example, exercise recommendation for heart failure states,

> *"Focus on low-impact activities, such as walking, cycling, swimming, or light resistance training, which can improve cardiovascular fitness without putting too much stress on the joints."*

In some instances, "low-impact" was used interchangeably with low-intensity. For example, exercise recommendations for older adults suggested,

> *"Aerobic exercise intensity: Aerobic exercise, also known as cardio, is any exercise that gets your heart rate up and increases your breathing. Good examples for older adults include walking, swimming, cycling, or low-impact aerobics."*

Theme 3: Bias / discrimination: A majority of AI-generated output was written following a standard format (i.e., conversational) and in the second-person tense with the exception of intellectual disability; older adults; and children and adolescents, which were provided in the third-person tense and using the "they/them" pronouns. For example, exercise recommendations for individuals with intellectual disability read as,

> *"Make it fun: It's important to make exercise fun and enjoyable for individuals with intellectual disabilities. Incorporate games, music, and other fun activities to keep them engaged."*

Similarly, an excerpt from the exercise recommendations for older adults read as,

“However, it's important for older adults to choose exercises that are appropriate for their age and fitness level, and to check with their doctor before starting a new exercise program.”

In addition, it is worth noting that 100% of exercise examples were activities that may only be performed by able-bodied individuals.

Theme 4: Type and modality preference: There was a strong bias towards aerobic exercise with major gaps in content for resistance [frequency (n=16), intensity (n=18), time (n=25)] and flexibility [frequency (n=22), intensity (n=17), time (n=24)]. In addition, there was an interesting emphasis and endorsement of tai chi as a modality to improve flexibility and/or balance (n=7 mentions); often times as a stand-alone exercise type. For example, exercise recommendations for fibromyalgia suggested,

“Tai Chi: Tai Chi is a gentle form of exercise that involves slow, flowing movements and deep breathing. It has been shown to improve balance, reduce pain, and improve overall quality of life in people with fibromyalgia.”

Theme 5: Role of the exercise professional: There was varying (and sometimes improper) terminology used in reference to the role of an exercise professional, including: “qualified exercise professional” (n=3), “certified exercise professional” (n=1), “licensed exercise physiologist” (n=1), and “exercise physiologist” (n=1). In addition, these terms were used interchangeably with “physical therapist” (n=3).
